# Factors associated with chronic and acute back pain in Wales, a cross-sectional study

**DOI:** 10.1186/s12891-019-2477-4

**Published:** 2019-05-15

**Authors:** Steinthora Jonsdottir, Haroon Ahmed, Kristinn Tómasson, Ben Carter

**Affiliations:** 1 Administration of Occupational Safety and Health, Dvergshofdi 2, 110 Reykjavik, Iceland; 2 Division of Population Medicine, Cardiff University School of Medicine, Heath Park, Cardiff, CF14 4YS UK; 3 Department of Biostatistics and Health Informatics, Institute of Psychiatry, Psychology & Neuroscience, King’s College London, De Crespigny Park, London, SE5 8AF UK

**Keywords:** Chronic Back pain, Acute back pain, Risk factors, Physical activity, Prevention

## Abstract

**Background:**

Back pain is one of the most common causes for disability in the working population. Some risk factors for back pain are well known, however little is known about factors uniquely associated with acute or chronic back pain.

This study aimed to elucidate patterns uniquely associated with acute or chronic back pain.

**Methods:**

This study performed secondary analysis of data from the Welsh Health Survey 2012, a nationwide cross-sectional survey.

A multivariable analysis was carried out for risk factors found to be significantly associated with acute and chronic back pain.

**Results:**

We found that increased BMI (aOR 1.20, 95% Cis 1.08, 1.33; BMI > 30), mental health score below average (aOR 1.59, 95% CIs 1.47, 1.72), having a degree (aOR 1.28, 95% CIs 1.12, 1.47) and being older than 24 years (*P* < 0.001) were associated with increased prevalence of acute back pain.

Higher prevalence of chronic back pain was seen in individuals characterised by increased deprivation (WIMD) (aOR 1.61, 95% CIs 1.32, 1.96); increased age (aOR 7.34, 95% CIs 5.25, 10.26; for 65+); being female (aOR = 1.43, 95% CIs 1.27, 1.61); lower educational attainment (aOR 0.44, 95% CIs 0.36, 0.55) higher BMI (aOR = 1.60 95% CIs 1.38, 1.85; BMI > 30); poorer mental health score (aOR = 3.11 95% CIs 2.76, 3.51), and a sedentary lifestyle (aOR = 0.58, 95% CIs 0.49, 0.69; 3–5 days of light exercise).

**Conclusion:**

Increased deprivation, female gender, and little exercise were uniquely associated with chronic back pain. These characteristics may help clinicians to intervene to prevent acute backpain resulting in chronic cases.

## Background

Back pain is a common and potentially disabling condition that can lead to reductions in quality of life, time off work and long-term disability. The Global Burden of Disease Study estimated the point prevalence of low back pain to be 9.4%, and reported low back pain to be the condition responsible for the most years lived with disability [1]. Back pain is one of the most common causes for disability in the working population, and severely impacts upon work productivity and absenteeism [1]. In the UK alone, almost 3.4 million working days were lost due to work-related back pain in 2016/17, that is 13.3% of all working days lost due to ill health [2]. Low back pain is the reason for one in every seven general practice consultations [3]. The associated health care cost and burden has been reported across health care systems worldwide [4, 5]. Hong et al. [6] found that the healthcare costs of patients suffering chronic low back pain (CLBP) were double those of matched controls without CLBP.

### Chronic and acute back pain

Back pain is defined as acute when it has persisted for up to 6 weeks and sub-acute when it has persisted for up to 3 months [7]. Chronic back pain is defined as back pain that is present for more than 3 months [8] and is associated with patients receiving treatment [9, 10]. Acute back pain is often the result of actual or near tissue injury or sprain [7] and individuals with acute back pain are less likely to seek care or be referred for treatment [9, 10]. Chronic pain often persists even though the initial injury has healed [7]. These cases are more likely to be referred for treatment than the more acute cases that are commonly left untreated [9, 10].

### Risk factors

There is good evidence for an association between increasing age and obesity (BMI > 30) and risk of back pain [4, 11–21] and that obesity is a strong predictor of disability caused by back pain [20, 22, 23]. It is also known that the prevalence and severity of back pain is higher where there is greater deprivation [4, 12, 14–16, 19–21, 24–28]. There is conflicting evidence on the effect of physical activity (PA) on back pain. Heneweer et al. [13] suggested a U-shaped dose-response relationship between PA and back pain. Other studies have found that physical inactivity is associated with a significant increase in risk of back pain [17, 22]. There is some evidence suggesting that females have a greater risk of back pain, [4, 11–21, 24] however a recent global study reported this varied by region [1].

There is limited evidence that job demands including lifting and twisting [13, 20, 26, 29]; ethnicity [18, 24]; genetic factors [14]; and mental health comorbidities [4, 14, 22, 26] are all associated with higher risk of back pain. The varying level of evidence, available literature and the lack of a standardised definition of back pain make definitive conclusions challenging [30, 31].

## Methods

### Aim and objectives

This study aimed to elucidate patterns uniquely associated with acute or chronic back pain. Differentiating between the two is challenging in clinical practice. Identifying risk factors associated with the pattern may help clinicians differentiate between the two conditions, manage them more appropriately and ultimately help to improve patient outcomes. In addition this could enable targeting of those at greatest risk for prevention through e.g. workplace modification strategies.

### Study design

We used a population based cross-sectional survey (The Welsh Health Survey 2012). The survey collected information on health status, illnesses, lifestyle and health service use in the general population. The sampling frame includes 99% of all private households in Wales. A sample of 14,775 households were drawn, stratified by geographical area. To achieve the aim of at least 600 interviews per geographical area, a minimum of 575 households were sampled in each geographical area. Household data were collected by enumerator from each adult aged 16 years or older. Further details about collection of data can be found on the Welsh Health Survey 2013 (WHS) [32].

### Outcomes

Primary outcomes in this study were:

- Acute back pain (episodes of untreated backache in the last 12 months) [9, 10]
- Chronic back pain (Back pain currently being treated) [9, 10]

For the purpose of this study back pain currently being treated was considered a measure of chronic back pain, and untreated backache in the last 12 months considered a measure of acute back pain.

### Covariates

The following mechanistically plausible covariates were investigated for associations with back pain (acute, and chronic):

- *Demographic*: Age (age bands 16–24, 25–44, 45–64, 65+); Gender.
- *Socioeconomic*: Educational attainment (No qualification, other qualification, degree equivalent or above); Occupational status (Managerial and professional, intermediate, routine and manual, never worked/long term unemployed); Welsh index of multiple deprivation 2014 (WIMD) (Deprivation quintiles).
- *Clinical*: Mental health measured by the SF-36 (< 50 vs. > 50); BMI (less than 18.5, 18.5–25, 25–30, 30 and over); Depression (treated vs. untreated); Anxiety (treated vs. untreated); Physical activity (PA) (meeting the UK PA guidelines vs. not meeting them and number of days of light, moderate or vigorous exercise per week).

### Data analysis

An a priori statistical analysis plan was followed (available on request). Descriptive statistics tabulated demographic and risk factors, for acute and chronic back pain, and counts were presented. Crude logistic regression models were fitted to each risk factor and odds ratios (ORs) were presented with 95% confidence intervals (95% CI) and *P*-values. A multivariable logistic regression model with a forward stepping approach where a likelihood ratio test (LRT) of sequential nested models, was used to determine parsimonious independent associations with the covariates (*p* < 0.01). The final analyses were inclusive of all risk factors from either of the analyses. The analysis was adjusted for the clustered nature of the respondents within geographical areas within the UK, by estimating inflated standard errors using the robust cluster estimators of the variances. Stata 13 was used for all analyses.

## Results

There were 19,282 eligible adults who were invited in the WHS 2013, and 15,007 were included in the analysis. The response rate was higher among women (83.1%) than men (79.4%), as well as among older individuals than younger individuals (70.3% for 16–24 years, 75.6% for 25–44 years, 85.1% for 45–64 years, 88.9% for 65 years and older). There was less than 5% missing data for any included variable.

The prevalence of acute back pain was 31.5% and the prevalence of chronic back pain was 13.4% (Table 1). The prevalence of reported acute *and* chronic back pain combined was 39.1%.

**Table 1:** Numbers and proportions of acute and chronic back pain across all covariates

	Acute back pain		Chronic back pain		All back pain	
	Total	Pain (%)	Total	Pain (%)	Total	Pain (%)
Total	14,359	4519 (31.5%)	14,351	1772 (13.4%)	14,100	5520 (39.1%)
Deprivation (WIMD quintile)	14,359		14,351		14,100	
Least deprived	2839	892 (31.42)	2859	248 (8.67)	1029	1029, (36.65)
2	3065	994 (32.43)	3053	347 (11.37)	1207	1207, (40.07)
3	3275	1053 (32.15)	3309	409 (12.36)	1275	1275, (47.05)
4	2762	866 (31.35)	2745	381 (13.88)	1072	1072, (45.79)
Most deprived	2418	714 (29.53)	2385	387 (16,23)	2529	2529, (108.03)
Age (years)	14,359		14,351			
16–24	1718	387 (22.53)	1752	46 (2.63)	410	410, (24.05)
25–44	3830	1264 (33.84)	3879	275 (7.09)	1421	1421, (37.25)
45–64	4963	1731 (34.88)	4974	709 (14.25)	2144	2144, (43.76)
65+	3848	1137 (29.55)	3746	742 (19.81)	1545	1545, (41.97)
Gender	14,359		14,351			
Female	7699	2480 (32.21)	7667	1084 (14.14)	3098	3098, (41.05)
Male	6660	2039 (30.62)	6684	688 (10.29)	2422	2422, (36.96)
Educational attainment	13,398		13,424			
No qualification	2643	752 (28.45)	2573	573 (22.27)	1077	1077, (42.27)
Other qualification	8367	2731 (32.64)	8443	883 (10.46)	3231	3231, (39.01)
Degree Equivalent and above	2388	774 (32.41)	2408	136 (5.65)	856	856, (36.03)
Occupational status (NS-SEC)	13,959		13,936			
Managerial and Professional occupations	5170	1605 (31.04)	5228	461 (8.82)	1868	1868, (36.52)
Intermediate occupations	2853	964 (33.79)	2834	330 (11.64)	1143	1143, (40.88)
Routine and manual occupations	5569	1718 (30.85)	5524	883 (15.98)	2217	2217, (40.74)
Never worked and long-term unemployed	357	104 (29.13)	350	65 (18.57)	143	143, (41.81)
BMI^a^	13,387		13,391			
Less than 18.5	281	59 (21.00)	284	21 (7.39)	71	71, (26.01)
18.5 to under 25	5176	1498 (28.94)	5213	490 (9.40)	1778	1778, (34.88)
25 to under 30	4878	1604 (32.88)	4873	594 (12.19)	1943	1943, (40.45)
30 and over	3052	1073 (35.16)	3021	550 (18.21)	1386	1386, (46.31)
Mental Health (SF-36 mental health score)	14,359		14,351			
Higher than average (> 50)^b^	8803	2421 (27.50)	8862	632 (7.13)	2783	2783, (32.15)
Lower than average (< 50)^c^	5556	2098 (37.76)	5489	1140 (20.77)	2737	2737, (50.28)
Depression	13,840		14,165			
Yes	1257	488 (38.82)	1183	403 (34.07)	717	717, (59.7)
No	12,583	3832 (30.45)	12,982	1204 (9.27)	4519	4519, (35.85)
Anxiety	13,776		14,124			
Yes	1025	392 (38.24)	959	315 (32.85)	568	568, (58.32)
No	12,751	3904 (30.62)	13,156	1252 (9.52)	4624	4624, (36.16)
Exercise	14,136		14,139			
Meeting PA guidelines^d^	4106	1291 (31.44)	4156	269 (6.47)	1432	1432, (35.33)
Not meeting guidelines	10,030	3164 (31.55)	9983	1451 (14.53)	4001	4001, (40.64)

^a^Body mass index

^b^Mental health score above the average of the general population

^c^Mental health score below the average of the general population

^d^Meeting physical activity guidelines of 30 min of light to moderate exercise on at least 5 days of the week

### Acute back pain

The crude analysis found that increased BMI (aOR 1.20, 95% CIs 1.08, 1.33; BMI > 30), mental health score below average (aOR 1.59, 95%CIs 1.47, 1.72; mental health score below avg), having a degree (aOR 1.28, 95% CIs 1.12, 1.47; Degree or higher) and being older than 24 years (*P* < 0.001) were associated with increased prevalence of acute back pain. In a multivariable analysis we found consistent results with the crude analysis (Table 2).

**Table 2:** Univariable logistic regression of acute back pain and multivariable logistic regression of acute back pain, adjusted for significantly associated covariates

	Univariable analysis			Multivariable analysis	
	N	Crude OR (95% CI)	*P* value	Adjusted OR (95% CI)	*P* value
WIMD 2014 quintile	14,359				
Least deprived		Reference category			
2		1.05 (0.94, 1.17)	0.405	1.03 (0.92, 1.16)	0.611
3		1.03 (0.93, 1.15)	0.539	1.00 (0.89, 1.13)	0.940
4		1.00 (0.89, 1.12)	0.958	0.93 (0.82, 1.05)	0.261
Most deprived		0.91 (0.81, 1.03)	0.138	0.86 (0.76, 0.98)	0.029
Age	14,359				
16–24		Reference category			
25–44		1.69 (1.49, 1.93)	< 0.001	1.64 (1.42, 1.90)	< 0.001
45–64		1.84 (1.62, 2.09)	< 0.001	1.73 (1.50, 1.99)	< 0.001
65+		1.44 (1.26, 1.65)	< 0.001	1.49 (1.27, 1.73)	< 0.001
Gender	14,359				
Male		Reference category			
Female		1.08 (1.00, 1.16)	0.040	1.01 (0.94, 1.10)	0.761
Educational attainment	13,398				
No qualification		Reference category			
Degree equivalent or higher		1.21 (1.07, 1.36)	0.002	1.28 (1.12, 1.47)	< 0.001
Other qualifications		1.22 (1.11, 1.34)	< 0.001	1.32 (1.18, 1.47)	< 0.001
BMI^a^	13,387				
Less than 18.5		0.65 (0.49, 0.87)	0.004	0.79 (0.58, 1.07)	0.127
18.5 to under 25		Reference category			
25 to under 30		1.20 (1.11, 1.31)	< 0.001	1.14 (1.04, 1.25)	0.004
30 and over		1.33 (1.21, 1.46)	< 0.001	1.20 (1.08, 1.33)	0.001
Mental health (SF-36)	14,359				
Above average^b^		Reference category			
Below average^c^		1.60 (1.49, 1.72)	< 0.001	1.59 (1.47, 1.72)	< 0.001
Vigorous exercise	13,757				
0–2 days per week		Reference category			
3–5 days		0.83 (0.73, 0.94)	0.03	0.91 (0.80, 1.03)	0.156
6–7 days		0.83 (0.68, 1.02)	0.077	0.91 (0.74, 1.13)	0.406

^a^Body mass index

^b^Mental health score above the average of the general population

^c^Mental health score below the average of the general population

### Chronic back pain

In the multivariable analysis higher rates of chronic back pain were seen in individuals who were characterised by increased deprivation (WIMD) (aOR 1.61, 95% CIs 1.32, 1.96; most deprived); increased age (aOR 7.34, 95% CIs 5.25, 10.26; for 65+); being female (aOR = 1.43, 95% CIs 1.27, 1.61); lower educational attainment (aOR 0.44, 95% CIs 0.36, 0.55; degree or higher) higher BMI (aOR = 1.60 95% CIs 1.38, 1.85; BMI > 30); poorer mental health score (aOR = 3.11 95% CIs 2.76, 3.51; below average), and a sedentary lifestyle (aOR = 0.58, 95% CIs 0.49, 0.69; 3–5 days of light exercise) (Table 3).

**Table 3:** Univariable logistic regression of chronic back pain and multivariable logistic regression of chronic back pain, adjusted for significantly associated covariates

	Univariable analysis			Multivariable analysis	
	N	Crude OR (95% CI)	P value	Adjusted OR (95% CI)	P value
WIMD 2014 quintile	14,351				
Least deprived		–	–	–	–
2		1.35 (1.14, 1.60)	0.001	1.32 (1.09, 1.60)	0.005
3		1.48 (1.26, 1.75)	< 0.001	1.33 (1.10, 1.61)	0.003
4		1.70 (1.43, 2.01)	< 0.001	1.42 (1.16, 1.72)	< 0.001
Most deprived		2.04 (1.72, 2.42)	< 0.001	1.61 (1.32, 1.96)	< 0.001
Age	14,351				
16–24		–	–	–	–
25–44		2.83 (2.06, 3.89)	< 0.001	2.42 (1.71, 3.42)	< 0.001
45–64		6.17 (4.55, 8.35)	< 0.001	5.14 (3.69, 7.15)	< 0.001
65+		9.16 (6.76, 12.41)	< 0.001	7.34 (5.25, 10.26)	< 0.001
Gender	14,351				
Male		–	–	–	–
Female		1.44 (1.30, 1.59)	< 0.001	1.43 (1.27, 1.61)	< 0.001
Educational attainment	13,424				
No qualification		–	–	–	–
Degree equivalent or higher		0.21 (0.17, 0.25)	< 0.001	0.44 (0.36, 0.55)	< 0.001
Other qualifications		0.41 (0.36, 0.46)	< 0.001	0.75 (0.65, 0.86)	< 0.001
BMI^a^	13,391				
Less than 18.5- Underweight		0.77 (0.49, 1.21)	0.258	0.91 (0.55,1.48)	0.699
18.5 to under 25- Normal weight		–	–	–	–
25 to under 30- Overweight		1.34 (1.18, 1.52)	< 0.001	1.20 (1.04, 1.38)	0.013
30 and over- Obese		2.15 (1.88, 2.45)	< 0.001	1.60 (1.38, 1.85)	< 0.001
Mental health (SF-36)	14,351				
Above average^b^		–	–	–	–
Below average^c^		3.41 (3.08, 3.79)	< 0.001	3.11 (2.76, 3.51)	< 0.001
Light exercise	14,014				
0–2 days per week		–	–	–	–
3–5 days		0.43 (0.37, 0.49)	< 0.001	0.58 (0.49, 0.69)	< 0.001
6–7 days		0.39 (0.35, 0.43)	< 0.001	0.55 (0.48, 0.63)	< 0.001

^a^Body mass index

^b^Mental health score above the average of the general population

^c^Mental health score below the average of the general population

In the crude analysis, all covariates were found predictive of chronic back pain. Increasing age and BMI were found to offer the greatest increase in odds of chronic back pain (Table 3).

## Discussion

The study aimed to describe a pattern of acute and chronic back pain and examine possible risk factors in order to elucidate differences between the sub-types of back pain. We found that increasing age, higher BMI, better educational attainment and poorer mental health were independently associated with both acute and chronic back pain. However, we also found that increasing WIMD quintile (i.e., increasing deprivation), female gender, and exercising less than 2 days per week were uniquely associated with chronic back pain.

This is the first population-based study to compare independent associations for acute and chronic back pain. The strength was larger for all of the associations for chronic back pain and the associations showed a diluted effect in acute back pain in most of the covariates.

### Comparison with existing literature

Educational attainment had the opposite effect on acute back pain compared to chronic back pain, and higher educational attainment was significantly associated with increased odds of acute back pain. Riskowski [33] reported a similar finding in a cross-sectional survey conducted in the U.S., in which they found that chronic back pain was more common in individuals of lower socioeconomic position and that acute back pain was more common in individuals of higher socioeconomic positions. Riskowski suggests that these unusual findings could be related to changes in socioeconomic positions over time as acute pain becomes chronic [33]. Assuming that untreated backache represents acute cases and treated back pain represents chronic cases similar suggestions might be made for this study, as educational attainment is an important marker for socioeconomic status and deprivation. Definitive explanations of these findings are difficult, although speculative suggestions can be made that cases of acute back pain in those with higher educational attainment are less likely to become chronic because of better knowledge of self-regulation or coping strategies in addition to this group having in general better means. This would result in most back pain cases in those with higher educational attainment being acute and not becoming chronic. We found obesity (BMI > 30) to be independently associated with chronic back pain, this is in line with previous studies [4, 11–21]. Fransen et al. (2002) found obesity to be a significant predictor of chronicity in individuals receiving compensation for working days lost due to acute back pain [34].

A recent systematic review found that stratified programmes were effective in preventing the development of chronic back pain. Those classified at low risk of developing chronic back pain benefited from simple educational messages while those classified at medium or high risk benefited from a combination of reactivation programmes, exercise and cognitive-behavioural interventions. We have identified factors independently associated with chronic back pain only. This may help to determine the risk of patients developing chronic back pain, and in turn determine a suitable prevention intervention [35].

Our findings in general are in line with previous studies however it is the first in the UK to distinguish between acute and chronic back pain.

### Strengths and limitations

This is the first population-based study of back pain in the UK, and the first to differentiate between acute and chronic back pain. The reported results cannot infer causality due to the nature of the study design. Multivariable analyses controlled for known confounders, however this doesn’t include the unknown confounders, i.e. work demands, chronic stress and genetic factors. There is a limitation in the measures for chronic and acute back pain used in this study. The evidence suggests that treated cases are likely to represent chronic cases and untreated cases are likely to represent acute cases [9, 10]. However, we anticipate that some cases may be misclassified, as acute back pain may sometimes be treated with for example, anti-inflammatories.

There is debate over these definitions and this is unlikely to be universal. Potential biases affecting the study include selection bias and reporting bias. We cannot ignore the possibility of reverse causality. Given the weaknesses, caution is needed when interpreting these findings, however, this study gives a clue about the difference in risk factors between acute and chronic back pain.

## Conclusion

Chronic back pain is a considerable public health concern and risk factors for acute and chronic back pain are different. This study has identified factors associated with chronic back pain that are not associated with acute back pain. This information may help clinicians to intervene to prevent acute back pain resulting in chronic cases. More emphasis should be put on service for those in deprived areas. In addition this information can help target groups and individuals for preventive measures.

Longitudinal cohort studies are needed to make conclusions about causality regarding risk factors of back pain and to distinguish successfully between cases that progress form acute to chronic. In addition further analysis of long-term cohort studies are needed to investigate the effect of light exercise on chronic back pain as a suggested means of self-management.

## Abbreviations

aOR: adjusted Odds Ratio
BMI: Body mass index
CLBP: Chronic low back pain
LRT: Likelihood ratio test
OR: Odds Ratio
PA: Physical activity
WHS: Welsh health survey
WIMD: Welsh Index of Multiple Deprivation

## References

[1] Hoy D, March L, Brooks P, Blyth F, Woolf A, Bain C (2014). The global burden of low back pain: estimates from the global burden of disease 2010 study. Ann Rheum Dis.

[2] Work-related Musculoskeletal Disorders (WRMSDs) Statistics in Great Britain 2017 [Internet]. 2017. Available from: www.hse.gov.uk/statistics/. Accessed 20 Feb 2018.

[3] Jordan KP, Kadam UT, Hayward R, Porcheret M, Young C, Croft P (2010). Annual consultation prevalence of regional musculoskeletal problems in primary care: an observational study. BMC Musculoskelet Disord.

[4] Langley PC (2011). The prevalence, correlates and treatment of pain in the European Union. Curr Med Res Opin.

[5] Dagenais S, Caro J, Haldeman S (2008). A systematic review of low back pain cost of illness studies in the United States and internationally. Spine J.

[6] Hong J, Reed C, Novick D, Happich M (2013). Costs associated with treatment of chronic low back pain: an analysis of the UK general practice research database. Spine (Phila Pa 1976).

[7] Goodwin J, Bajwa ZH. Understanding the patient with chronic pain. In: Warfield CA, Bajwa ZH, editors. Principles & Practice of Pain Medicine, 2nd Edition. New York: McGraw-Hill; 2004. ISBN: 0-07-144349-5

[8] van Tulder M, Becker A, Bekkering T, Breen A, del Real MTG, Hutchinson A (2006). Chapter 3 European guidelines for the management of acute nonspecific low back pain in primary care. Eur Spine J.

[9] Ferreira ML, Machado G, Latimer J, Maher C, Ferreira PH, Smeets RJ (2010). Factors defining care-seeking in low back pain - a meta-analysis of population based surveys. Eur J Pain.

[10] Fischbein R, McCormick K, Selius BA, Labuda Schrop S, Hewit M, Baughman K (2015). The assessment and treatment of back and neck pain: an initial investigation in a primary care practice-based research network. Prim Heal Care Res Dev.

[11] Breivik H, Collett B, Ventafridda V, Cohen R, Gallacher D (2006). Survey of chronic pain in Europe: prevalence, impact on daily life, and treatment. Eur J Pain.

[12] Volkers AC, Westert GP, Schellevis FG (2007). Health disparities by occupation, modified by education: a cross-sectional population study. BMC Public Health.

[13] Heneweer H, Vanhees L, Picavet H (2009). Physical activity and low back pain: a U-shaped relation?. Pain..

[14] van Hecke O, Torrance N, Smith BH. Chronic pain epidemiology and its clinical relevance. Br J Anaesth. 2013;111(1):13–18. 10.1093/bja/aet123 23794640

[15] Bergman S, Herrstrom P, Hogstrom K, Petersson IF, Svensson B, Jacobsson LT (2001). Chronic musculoskeletal pain, prevalence rates, and sociodemographic associations in a Swedish population study. J Rheumatol.

[16] Dorner TE, Muckenhuber J, Stronegger WJ, Rsky É, Gustorff B, Freidl W (2011). The impact of socio-economic status on pain and the perception of disability due to pain. Eur J Pain.

[17] Jhun HJ, Park JY (2009). Estimated number of Korean adults with back pain and population-based associated factors of back pain: data from the fourth Korea National Health and nutrition examination survey. J Korean Neurosurg Soc.

[18] Johannes CB, Le TK, Zhou X, Johnston JA, Dworkin RH (2010). The prevalence of chronic pain in United States adults: results of an internet-based survey. J Pain.

[19] Latza U, Kohlmann T, Deck R, Raspe H (2004). Can health care utilization explain the association between socioeconomic status and back pain?. Spine (Phila Pa 1976).

[20] Leclerc A, Gourmelen J, Chastang J-F, Plouvier S, Niedhammer I, Lanoë J-L (2009). Level of education and back pain in France: the role of demographic, lifestyle and physical work factors. Int Arch Occup Environ Health.

[21] Schmidt CO, Raspe H, Pfingsten M, Hasenbring M, Basler HD, Eich W (2007). Back pain in the German adult population: prevalence, severity, and sociodemographic correlates in a multiregional survey. Spine (Phila Pa 1976).

[22] Leino-Arjas P, Hänninen K, Puska P (1998). Socioeconomic variation in back and joint pain in Finland. Eur J Epidemiol.

[23] Tokuda Y, Ohde S, Takahashi O, Shakudo M, Yanai H, Shimbo T (2007). Musculoskeletal pain in Japan: prospective health diary study. Rheumatol Int.

[24] Webb R, Brammah T, Lunt M, Urwin M, Allison T, Symmons D (2003). Prevalence and predictors of intense, chronic, and disabling neck and back pain in the UK general population. Spine (Phila Pa 1976).

[25] JL C, JA M (2005). The impact of social deprivation on chronic back pain outcomes. Chronic Illn.

[26] Moffett JA, Underwood MR, Gardiner ED (2009). Socioeconomic status predicts functional disability in patients participating in a back pain trial. Disabil Rehabil.

[27] Hagen KB, Holte HH, Tambs K, Bjerkedal T (2000). Socioeconomic factors and disability retirement from back pain. A 1983-1993 population-based prospective study in Norway. Spine (Phila Pa 1976).

[28] Hagen K, Zwart JA, Svebak S, Bovim G, Jacob Stovner L (2005). Low socioeconomic status is associated with chronic musculoskeletal complaints among 46,901 adults in Norway. Scand J Public Heal.

[29] Picavet HSJ, Schuit AJ (2003). Physical inactivity: a risk factor for low back pain in the general population?. J Epidemiol Community Health.

[30] Sitthipornvorakul Ekalak, Janwantanakul Prawit, Purepong Nithima, Pensri Praneet, van der Beek Allard J. (2010). The association between physical activity and neck and low back pain: a systematic review. European Spine Journal.

[31] Rossignol M, Rozenberg S, Leclerc A (2009). Epidemiology of low back pain: What’s new?. Joint Bone Spine.

[32] Sadler K, Doyle M, Hussey D, Stafford R. Welsh Health survey 2012: technical report [internet]. 2013. Available from: http://doc.ukdataservice.ac.uk/doc/7459/mrdoc/pdf/7459_technical_report.pdf. Accessed 3 July 2015.

[33] Riskowski JL (2014). Associations of socioeconomic position and pain prevalence in the United States: findings from the National Health and nutrition examination survey. Pain Med.

[34] Fransen M, Woodward M, Norton R, Coggan C, Dawe M, Sheridan N (2002). Risk factors associated with the transition from acute to chronic occupational back pain. Spine..

[35] Meyer C, Denis CM, Berguin AD (2018). Secondary prevention of chronic musculoskeletal pain: a systematic review of clinical trials. Ann Phys Rehabil Med.

